# Elderly and visually impaired indoor activity monitoring based on Wi-Fi and Deep Hybrid convolutional neural network

**DOI:** 10.1038/s41598-023-48860-5

**Published:** 2023-12-18

**Authors:** K. Deepa, Nebojsa Bacanin, S. S. Askar, Mohamed Abouhawwash

**Affiliations:** 1 Department of Computer Science and Engineering, K.Ramakrishnan College of Technology, Trichy, 621112 India; 2 Singidunum University, Belgrade, Serbia; 3 Department of Statistics and Operations Research, College of Science, King Saud University, P.O. Box 2455, 11451 Riyadh, Saudi Arabia; 4 Department of Computational Mathematics, Science and Engineering (CMSE), College of Engineering, Michigan State University, East Lansing, MI 48824 USA; 5 Department of Mathematics, Faculty of Science, Mansoura University, Mansoura, 35516 Egypt

**Keywords:** Computational biology and bioinformatics, Health care

## Abstract

A drop in physical activity and a deterioration in the capacity to undertake daily life activities are both connected with ageing and have negative effects on physical and mental health. An Elderly and Visually Impaired Human Activity Monitoring (EV-HAM) system that keeps tabs on a person’s routine and steps in if a change in behaviour or a crisis might greatly help an elderly person or a visually impaired. These individuals may find greater freedom with the help of an EVHAM system. As the backbone of human-centric applications like actively supported living and in-home monitoring for the elderly and visually impaired, an EVHAM system is essential. Big data-driven product design is flourishing in this age of 5G and the IoT. Recent advancements in processing power and software architectures have also contributed to the emergence and development of artificial intelligence (AI). In this context, the digital twin has emerged as a state-of-the-art technology that bridges the gap between the real and virtual worlds by evaluating data from several sensors using artificial intelligence algorithms. Although promising findings have been reported by Wi-Fi-based human activity identification techniques so far, their effectiveness is vulnerable to environmental variations. Using the environment-independent fingerprints generated from the Wi-Fi channel state information (CSI), we introduce Wi-Sense. This human activity identification system employs a Deep Hybrid convolutional neural network (DHCNN). The proposed system begins by collecting the CSI with a regular Wi-Fi Network Interface Controller. Wi-Sense uses the CSI ratio technique to lessen the effect of noise and the phase offset. The t- Distributed Stochastic Neighbor Embedding (t-SNE) is used to eliminate unnecessary data further. The data dimension is decreased, and the negative effects on the environment are eliminated in this process. The resulting spectrogram of the processed data exposes the activity’s micro-Doppler fingerprints as a function of both time and location. These spectrograms are put to use in the training of a DHCNN. Based on our findings, EVHAM can accurately identify these actions 99% of the time.

## Introduction

Statistical analysis of the global total shows that the proportion of the population that is 65 and up is growing at an alarming rate. The World Health Organization predicts that by 2050, 16% of the global population will be 65 or older. The Madrid World Action Plan on Ageing has highlighted numerous key approaches, including “making sure facilitating and supporting settings,” to welcome this demographic change and to prepare for the social reform it entails. Creating senior-friendly homes where people may remain safe, healthy, and independent for as long as possible is a top concern, which is why this directive was issued. For this reason, it is crucial to create reliable, inconspicuous, and geriatric-friendly in-home surveillance systems connected to a Health Information System (HIS) and programmed to summon help from a local emergency healthcare provider immediately. The foundation of every home monitoring system is human activity recognition (HAR). Human activity recognition (HAR) often involves making sense of sensor data to identify specific types of human behaviour. Data sensing, analysis, and classification components make up the bulk of a typical HAR system. The self-aware has a sensor or sensors that record data while the user performs various tasks. In most cases, raw sensor data is cleaned up by a data processing module before being sent to a classification one. The classification module categorises the actions, which employs a learning algorithm.

The use of wearable sensors^1^ in medicine is increasingly important in the current context^2^. The size and price of various wearable sensors have recently decreased, making them ideal for monitoring physical and recreational events, surveillance, interpersonal behaviour, physiotherapy, and the surveillance of elderly people to improve Ambient living^3–5^. Because it is the Internet of Things-based and, on the other hand, doesn’t need individual precautions, this would be a huge step forward in terms of security^6^. Although there has been a lot of attention to wearable digital monitors for weight, pulse rate, energy, and activity levels, few features realise or understand the difference between pharmaceutical-grade surveillance and effectively recording information^7^. IoT has just unveiled its iPhone well-being app, which will work similarly to the Healthcare Kit Development Platform, collecting health data and other apps^8^. Different types of health sensing techniques will be available for application developers to use. They collect data using connected and wireless sensors^9^. The wearable health app users will have access to their encrypted medical files on the internet and can communicate them with their doctors, clinics, and family members as necessary.

Human activity recognition (HAR) has been investigated for decades, but many concerns remain^9^. HAR intends to recognise physiological activities by analysing sensor and camera data. Thus, it can predict and prevent risky situations by revealing user patterns. HAR offers numerous system construction and deployment choices. Human activities are neither categorised nor defined generally. Second, there are many human activities. Thus, sensor placement and selection are crucial for distinguishing specific behaviours^10^. Thus, two critical challenges are selecting sensor measurements and collecting data in realistic conditions^9^. The HAR issue cannot be solved deterministically since sensor readings and user activities are diverse^9^. HAR systems increasingly use machine learning to detect human activity in sensor readings.

The need for an elderly monitoring system is increased because it saves time and money for people due to the early detection of hazards around them. The technology is upgraded to monitor the events at home, though we are outside. Safety is the primary concern for the elderly and visually impaired. These individuals are often more susceptible to accidents, falls, and other hazards due to limited mobility and impaired vision. Monitoring their activities can help prevent accidents and provide immediate assistance when needed. It allows for early detection of health issues, changes in behaviour, or signs of distress, enabling prompt medical intervention or adjustments to care plans. By assisting with daily activities and addressing potential safety concerns, monitoring activities improves the overall quality of life for the elderly and visually impaired. It allows them to enjoy more comfort and peace of mind. This monitoring technique can help reduce healthcare costs by preventing hospital admissions or nursing home placements. It will enable individuals to age in place, often more cost-effective than institutional care.

In RF-based systems, channel state information (CSI) data gathered by commercial Wi-Fi equipment is used to identify human actions. Structures usually on CSI are less expensive than radar systems and are more reliable than RRSI devices. Thanks to the widespread availability of Wi-Fi hotspots, CSI-based systems may be deployed extensively and cheaply. An innovative software solution for collecting CSI data from the network interface card is proposed in^11^. More than 400 investigations have made use of this instrument for a wide range of purposes, including detection and tracking^12^, indoor geolocation^13^, and motion tracking^14^. We create a radio-frequency (RF) sensor platform and gather CSI data throughout a building while a single person walks, trips, and sits. To lessen the influence of background noise on the CSI data, we employ several signal-processing methods. We build an activity recognition system using a deep learning framework and design feature extraction methods. We show that, on average, deep learning systems can attain a 99% accuracy rate. Compared to other activity recognition systems, ours performs better than the findings suggest.

The article is structured as follows: in Section "Related works", various relevant works are given, and in Section "Proposed methodology", the proposed system is explained, including specifics on the Wi-Fi wearable device, the selection of the neural network architecture, and the refinement of its performance. After that, in part IV, the experimental setup of the proposed model is discussed, and in Section "Result and discussion", specific tests are presented, and the results are talked about to validate the system. In the final portion, VI, we will draw some conclusions.

## Related works

The computer models used in the deep learning method are built up of several layers of processing power. Thus, the inherent structure in complicated and extensive datasets may be automatically learned. Deep learning is commonly utilised to complete tasks in healthcare using data collected by mobile systems^15^. The authors of^16^ explain how mobile devices and wearables paired with sensors are revolutionising health monitoring. There is a lot of potential for these gadgets to collect analytical data on many people, and deep learning is seen as a crucial part of analysing this new kind of data. However, there is still room for improvement in applying deep learning in healthcare sensing, primarily because of hardware limitations. Instead of analysing feature extractors from time-series sensor signals, the authors of^17^ argue that Deep Convolutional Neural Networks (DCNN) can gain knowledge of the discriminant features instantaneously for activity classification by using an activity image constructed from signal sequences from accelerometers and gyroscopes. Compared to the state-of-the-art, their outcomes on three available datasets were superior.

While previous works have discovered that some iterative aspects can accomplish well in recognising one action but poorly for others, in^18^ a Convolutional Neural Network (CNN) is used to perform the HAR job competently, extracting human activity highlights all with no technical experience (such as kitchen tasks or walking or running, walking, etc.). They highlight how a convolutional neural network (CNN) approach may successfully record variations of the same activity by feature extraction^19^ that are both local to the signals and spectrum. This system is also evaluated on three publicly available datasets, with the best accuracy of 96.88% being achieved by the researchers. The convolutional neural network (CNN) utilised in^1,20^ completes a HAR job with input from a single altimeter, allowing for constructing an angular velocity HAR on the mobile platform without needing specialised hardware. Using an Android app to capture tri-axial accelerometer data from participants, the findings reveal a pretty excellent accuracy of 93.8%. To preserve data variety, the studies were repeated with the device implanted in three other locations on the body. When compared to different prominent classifiers on the same dataset, such as the Support Vector Machine (SVM), CNN appears to have retrieved more valuable features than the manually computed input features of the Fast Fourier Transform (FFT) and Discrete Cosine Transform (DCT) used by the SVM^21^.

Previous research on these technologically advanced wheelchairs uses additional including heart rate, hypertension, glucose level, respiration rate, son and human actions, obstacle recognition, and movement^22,23^. Cushioned wheelchairs have pressure sensors that detect the user’s changing body position. When a sensor recognises a potentially harmful position, an alert sounds. For a wheelchair with a pressure sensor mattress, sensor readings are optimised, and then many classifiers are used on the information to choose the optimal classifier. In addition, daily actions, including stair climbing, chair climbing, standing still, and jogging, were detected using a revised modular support vector machine^24^. Detecting walking behaviours in raw data through the wavelet transform and the K closest neighbour classifier^25^ was also accomplished. Modern society places great value on human activity recognition (HAR). Yet, much work hasn’t been done to tackle the difficulty of classifying time-series data. Still, human activity detection using integrated smartphone sensors is a promising new avenue of study^26^. The author^27^ introduces the Spatiotemporal cRoss (STAR)-transformer to adequately express two cross-modal characteristics as an identifiable vector. Keyframes are first produced as global matrix tokens and skeletons as associated map tokens from the input video and skeleton sequence. After being compiled into multi-class tokens, these are fed through the STAR transformer.

In Garcia et al.^19^, a new HRC idea is presented, consisting of an HRC framework for controlling assembly operations carried out either in tandem with people and robots or independently by either group. When managing the setup, an HRC architecture that uses deep learning techniques needs only one piece of RGB camera data to create forecasts about the cooperative workplace and human behaviour^28^. The article^29^ collects features for ages 60 and above manually for analysis. The used feature fusion technology recognises the activity accurately. Here, no automated techniques of data collection are done. In Liang et al.^30^, radar-based data collection is done for HAR. This radar is nonwearable and senses the activity by fixing radar in mobile robots from a specific distance. The central issue is if the person moves beyond a specific reach, then the accuracy of the data is not assured. Also, sometimes it may miss the situation of serious fall down. The article recognises human activities using sensors at all time intervals. This sensor uses some convolution operation for sensing. However, real-time solid monitoring is not ensured. To overcome the sensing problem, this research uses Wi-sense technology for monitoring HAR using video recording and image capturing techniques. Further, a Hybrid deep learning model is used to access and process the images.

Opportunistic scheduling is a technique used in communication networks to maximise the utilisation of available resources (such as bandwidth or relay nodes) by selecting the best opportunities for data transmission^31–33^. It employs Model Predictive Control techniques to optimise security responses to enhance networked systems’ overall security and resilience in the face of cyber threats^34,35^. This optimisation is done while considering and mitigating the potential interference or mutual coupling effects between adjacent antenna elements^36^. This method analyses the similarity of paths and uses matrix algebra as part of its computational approach. Link prediction in directed networks is relevant in various fields^37–39^. It suggests that deep learning techniques are being applied to mitigate security risks and improve the overall security posture of IoT ecosystems. It is crucial as IoT devices become more integrated into our daily lives and various industries^40–42^. This research is relevant in modern wireless communication networks and the increasing demand for reliable and secure data transmission in various applications^43,44^. The system’s purpose is not just image analysis but also clinical evaluation, which means it aims to provide medical assessments and diagnoses based on these images^45,46^. Deep neural networks are employed to perform the task of matching or tracking features within soft tissues. Deep learning is a subset of artificial intelligence that excels at recognising patterns in data, making it suitable for tasks like image analysis and tracking^47–49^. This system utilises a specific microcontroller (STM32) for precise control of laser pulses, and it incorporates a Photomultiplier Tube (PMT) with adjustable gain to enhance the sensitivity of echo detection^50,51^. The Generalized buffer algorithm is a versatile approach to managing and controlling data or processes, providing a flexible solution that can be adapted to different situations where buffering is necessary for efficient operation^52,53^. The technology described involves the development of a framework that employs machine learning to optimise the entire communication process seamlessly within a system that integrates fibre-optic and terahertz communication technologies^54,55^. This information could be significant for neuroscience and clinical applications, as it may provide insights into the potential therapeutic or research applications of tACS for modulating neural activity in deeper brain structures^56–58^. It uses a structured hierarchical semantic network to represent and organise technological concepts or domains. Then, it employs dual-link prediction techniques to identify and assess potential connections or relationships within this network^59^.

## Proposed methodology

**Figure 1:**
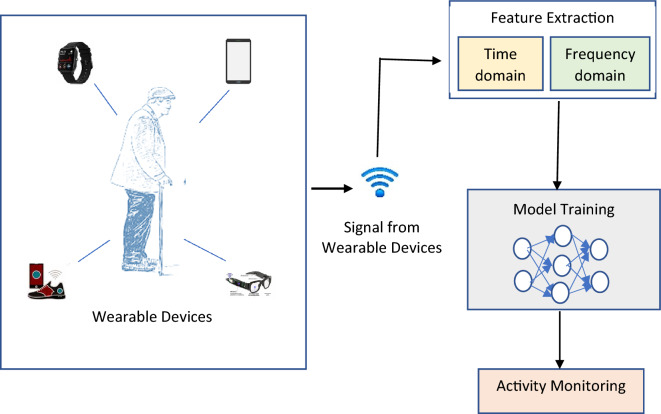
EV-HAM—model architecture.

At the outset, a broad range of sensors captures raw acoustic inputs (smartphones, Wi-Fi, watches, Bluetooth, sound etc.). Figure 1 shows an overview of a popular Pattern Recognition approach that may be used to deal with HAM. Second, when using deep learning techniques, attributes are derived from the readings. Some examples of these parameters include the average, the range, the DC, and the intensity. Lastly, such features are utilized as inputs for learning a PR model that can detect activity in actual HAR activities. This is an important step since it ensures that the model is accurate.

### Wearables and sensors

Rest, moving, lying down, climbing, running, and jumping are all considered physical pursuits. More and more research is linking regular physical exercise to a lower risk of numerous chronic illnesses, including overweight, diabetes, and cardiovascular events, as well as improved mental health of the elderly. There is a wealth of information, such as activity length and intensity, captured by wearable devices during these activities, which might provide light on a person’s daily routine and health status. Dedicated solutions like Fitbit, for instance, may measure and record energy expenditure on smart devices. This can be a significant first step in monitoring physical activity and avoiding the onset of chronic illnesses. More than that, studies have found a link between how people get around (car, foot, bike, and public transportation) and weight gain. Users can benefit from more exercise and an improved understanding of their diseases if doctors can access data on their daily movement and transportation habits. Therefore, one of the most pervasive uses of HAR technologies has the chance to benefit significantly from incorporating smartwatches into the fitness and leisure industries.

Mobile phones, wristbands, spectacles, bands, gloves, bracelets, pendants, sneakers, and E-tattoos are just a few commercially available or prototype smart gadgets currently under demand. Overall, these gadgets are designed to be worn by a person from head to toe. Miniaturizing and lightening wearables have been made possible by developments in micro-electro-mechanical system new tech (light microscope gadgets, encompassing a centralized system such as a microcomputer and involved so that engages with the environment, such as microelectronics), which in turn reduces the barrier to entry for the widespread adoption of smartwatches and Networking technologies. The goal of HAR is to better human psychology so that computers can more intelligently anticipate and meet the needs of their users. To use the formal terminology, let’s say the user is engaging in activities that fall within the category of “activity set X.”

1 $$X={\{{x}_{z}\}}_{z=1}^{N}$$

The number of activities is denoted by $N$. A series of sensor readings records the action in,

2 $$R=\{{r}_{1},{r}_{2},{r}_{3},{r}_{4}.........{r}_{n}\}$$

The sequence activity is identified by the deep learning model,

3 $$\widehat{X}={\{\widehat{X}\}}_{y=1}^{N}=M(R)\quad \widehat{X}\in X$$

The actual activity from the dataset is referred as,

4 $${X}^{*}={\{{X}^{*}\}}_{y=1}^{N}=M(R)\quad {X}^{*}\in X$$

### Wi-Fi sensing module

The goal of the radio frequency (RF) section is to glean information about the channel’s time-dependent qualities brought on by human action. In this context, environmental transmission of RF signals occurs at predetermined frequencies and phases. The ambient receivers will then record these transmissions. The captured signals provide insight into the RF channels and the ways in which they change over time as a result of human activity. By analyzing the data collected, scientists may learn what kinds of activities are taking place in a certain area. The frequency domain and the time domain are the two most used measurement settings for wideband channels, respectively. Stepped frequency sweeping is used to take measurements in the frequency domain of a channel at a variety of tones within a specified bandwidth. The Vector Network Analyzer (VNA) calculates the ${K}_{l}$ variable to determine the channel’s complicated sound quality. Depending on the value of the ${K}_{l}$ parameter, the network’s transfer function is calculated as,

5 $$W\left(\alpha \right)\propto {K}_{l}\left(\alpha \right)$$

6 $$\alpha =(t,{f}^{\prime})$$

Using the VNA’s trigger resonant frequency, we get ${K}_{l}\left(t,{f}^{\prime}\right)\propto {K}_{l}(t,{f}^{\prime})$ where ${f}^{\prime}$’ is one of the VNA’s trigger frequencies.

Received Signal Strength Indicator (RSSI) and Channel State Information (CSI) are typically the two measurements that are used in Wi-Fi-based HAR approaches to obtain insight into the channel and the influence of human activities. However, if we collect the channel impulse response (CIR), we can supply far more information than we could with either RSSI or CSI alone. CIR can provide us with facts on the RF channel and the changes that occur to it as a result of the atmosphere and the actions of humans. Each CIR element data is stored on the wireless channel modeling from the transmitter to the receiver. This channel may be described as “the path that—strategic from the to the receiver.”

7 $${g=e}^{-i(2\pi {f}_{c}{d}_{k}[n]+{\theta }_{k})}$$

8 $$f(n;d)=\sum_{k=1}^{N}{A}_{k}(n)g\delta (d-{d}_{k}[n]), n\in \{0,......n-1\}$$

where $n$ represents the total number of observations, $N$ represents the number of constructive interferences, ${A}_{k}$ and $d$ represent the intensity and latency of the $N$-th scatterer respectively, ${f}_{c}$ represents the centre frequency, and ${\theta }_{k}$ represents the arbitrary beginning stage. If this were the case, the spanned rate would be,

9 $${fr}^{\prime}\in \left\{k(\delta f)|\left[\frac{M}{2}\right]<k<\left[\frac{M}{2}\right]\right\}$$

The noise present in the CSI data is first efficiently reduced by the data processing module of the Wi-Fi module. Following that, the spectrogram approach is used to extract time-variant micro-Doppler signals. The time-variant Doppler characteristics of the RF channel are shown as pictures on the spectrogram. These characteristics are generated by both stationary and moving objects. Because only moving things in the environment may produce the Doppler effect, we know dispersed signal components received from stationary objects will not experience any Doppler shift. This is because the Doppler effect is only created by moving things. Therefore, we contend that the fluctuations in the micro-Doppler signatures are caused by the moving item. As a result, the functionality of the Wi-Fi module will not be impacted by the positioning of various static objects. The categorization module receives spectrogram pictures that have been saved in JPEG format after being processed. The Wi-Fi categorization module is essentially a Hybrid CNN that analyses user behavior to categorize the various tasks that the user carries out.

### Hybrid convolutional neural network

**Figure 2:**
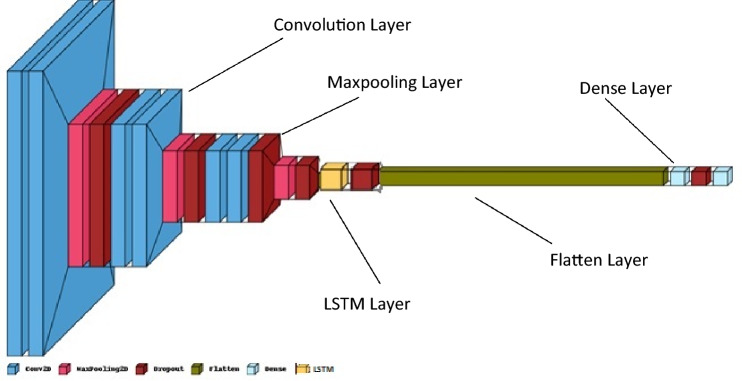
The DHCNN model architecture.

A CNN, an LSTM, attention, and a dense network comprise the human activity recognition network (Deep Hybrid CNN(DHCNN)), shown in Fig. 2. A dense network is used to recognize the behaviors of the subject. This network acts as a classifier by employing the residual concatenation for classification, which is then followed by CNN, long short—term memory, and the attention model. The suggested CNN-LSTM structure with self-attention model is depicted in Fig. 2. This framework makes use of CNN layers to dynamically extract attributes from information, and it also combines LSTMs and an attention layer to assist with sequence predictions. CNN-LSTMs equipped with self-attention are utilized in the production of textual files from captured images as well as the solution of difficulties involving the predicting of optical time series. This architecture is useful for addressing issues that call for the development of periodic output or that entail time and space input structures. In this study, a deep CNN-LSTM model that incorporates self-attention is proposed as a means of improving recognition accuracy.

10 $${a}_{k,l}=g\left(\sum_{a=1}^{X}\sum_{b=1}^{Y}{w}_{k,l}.{I}_{k+a,l+b}+B\right)$$

${a}_{k,l}$ represents the activation function. ${w}_{k,l}$ is the weight function. ${I}_{k+a,l+b}$ denotes the previous neuron and $B$ denotes the bias function. In the experimental that we ran, the deep networks used rectified linear units (ReLU) to compute the local features. The non-linear variable was represented using the following notation:

11 $$R(I)=\mathrm{max}(o,I)$$

In general, it has been found through examination that the more convolution kernels that are utilised, the more concealed characteristics of the input samples may be retrieved. One convolutional layer is present in the CNN-LSTM model that incorporates self-attention. In this convolution layer, there are a total of 16 kernels that are utilised for the feature extraction. The size of each convolutional kernel ranges from 1 to 5. At this point in time, LSTM networks function wonderfully across a broad spectrum of temporal schemes. The Long Short-Term Memory (LSTM) Recurrent Neural Network (RNN) is a form of RNN that is gaining more and more prominence. RNNs are able to make a forecast of the current time output based on the DL method’s reliance on prior information. However, because to the dissolving gradient issue, RNN systems can only recognize data for a limited amount of time at a time. If gradient are not permitted to flow deeply whereas the back-propagation approach is being used for deep learning, this will result in the gradients being buried. The RNN group was presented with a novel neuron that they came up with and named LSTM to solve the issue of long-term reliance.

In order to efficiently extract the temporal characteristics included within the sequence data, the authors of this paper begin by running the input data through a two-layer LSTM network. In the LSTM layer, there are a total of 64 memory cells. The action of each LSTM unit can be manipulated by using the given equations, which involves delivering a variety of inputs to a variety of gates, including input gates, exit gates, and entrance inputs.

**Algorithm 1 (DHCNN)**

*Step 1* Gather and preprocess your dataset of human activity data. This dataset should include both input data (e.g., images, sequences) and corresponding labels (e.g., activity categories).

*Step 2* Import the necessary libraries and deep learning frameworks such as TensorFlow, PyTorch, or Keras.

*Step 3* Define the architecture of the DHCNN model, including the following components:

o Input layer: Define the input shape based on your data.
o CNN layers: Specify the number of convolutional layers, filter sizes, activation functions, etc.
o LSTM layers: Specify the number of LSTM layers, the number of memory cells, return sequences if needed.
o Attention mechanism: Define the self-attention mechanism.
o Classifier (Dense network): Specify the dense layers for classification.

*Step 4* Create Individual Components

- Define functions to create individual components of the model:
  o create_cnn_layers: Define CNN layers.
  o create_lstm_layers: Define LSTM layers.
  o apply_self_attention: Define the self-attention mechanism.
  o create_dense_classifier: Define the dense layers for classification.

*Step 5* Create and compile the DHCNN model using your chosen deep learning framework. Compile it with an appropriate optimizer, loss function, and evaluation metrics.

*Step 6* Split your dataset into training, validation, and test sets. Typically, an 80–10-10 or 70–15-15 split is used.

*Step 7* Train the DHCNN model on the training data using the fit method. Specify the number of epochs, batch size, and validation data.

*Step 8* Evaluate the trained model on the test set to assess its performance. Calculate metrics like accuracy, precision, recall, and F1-score.

*Step 9* Fine-tune the model by adjusting hyperparameters, architecture, and regularization techniques to improve performance.

*Step 10* Once you are satisfied with the model’s performance, deploy it for inference on new, unseen data. You can deploy it as part of a larger application or system.

*Step 11* Continuously monitor the model’s performance and retrain it with new data or fine-tuning as necessary to maintain its accuracy.

### Ethical approval

None of the authors’ experimented with human subjects or animals during this research.

## Experimental setup

The scikit learn and Keras libraries on top of a tensor flow backend are used to train the model’s classification algorithms. The training and testing data is split as 80% and 20%.

### Dataset

The activity of the various areas of the human body causes changes in the reflectors of the wireless signals, which in turn results in variations in the CSI. People’s behaviour may be detected by conducting an analysis of the data streams produced by CSIs for various activities and correlating those streams of data to models that have been stored. This is accomplished by the extraction of features from CSI data streams and the application of machine learning techniques in the construction of models and classifiers.

It is necessary to have a dataset on hand in order to construct and train a model for human activity recognition. We discovered two datasets that are accessible to the public:^60,61^. Both sets of data were gathered by utilising the Linux 802.11n CSI Tool, and each of the transmitter and receiver routers had three antennae. Despite this, we made the decision not to utilise them because the hardware is now outdated and cannot be purchased elsewhere. In addition to this, the data collection was done in sequences, each of which consisted of only a single action.

**Figure 3:**
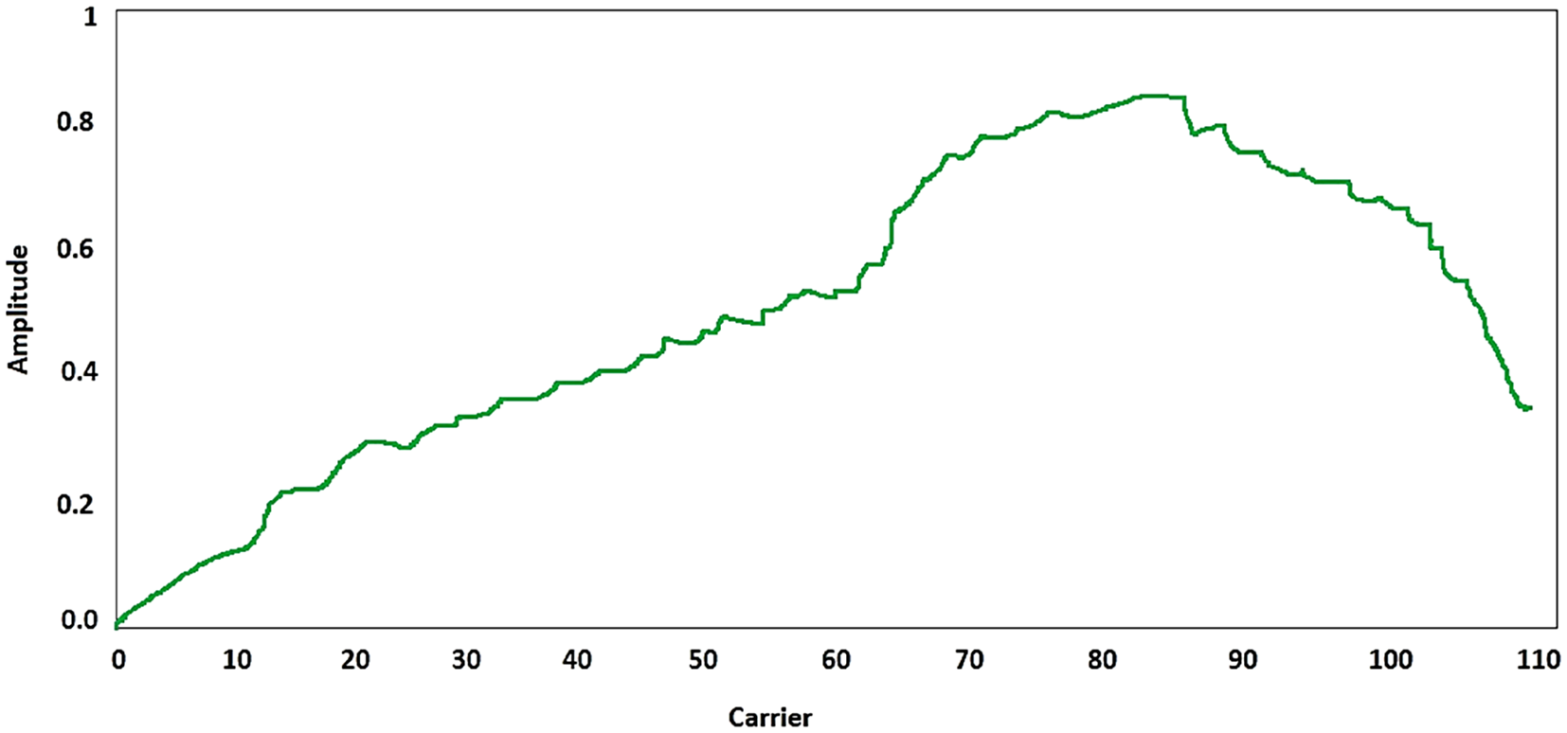
The amplitude collected from the dataset.

The series can be relatively lengthy in terms of time, but they do not include transitions between the many acts that take place, nor do they include actions that change rapidly and often over brief intervals of time. Due to these restrictions, a realistic depiction of human behaviour and data from the actual world is not possible. Last but not least, the setting in which the data is collected is one that is highly regulated and resembles a laboratory. The publicly available dataset is used in our model is shown in the Table 1 and the amplitude is shown in the Fig. 3. The total train and test data in this dataset is 1,801,440.

**Table 1:** Number of activities from the public dataset.

Id	Activity	Count
01	Jumping in place	142
02	Jumping jacks	186,510
03	Bending—hands up all the way down	445,011
04	Punching (boxing)	237,956
05	Waving—two hands	243,080
06	Waving—one hand (right)	254,089
07	Clapping hands	125,896
08	Throwing a ball	86,034
09	Sit down then stand up	498,088
10	Sit down	85,974
11	Stand up	69,164
12	T-pose	27,598

**Figure 4:**
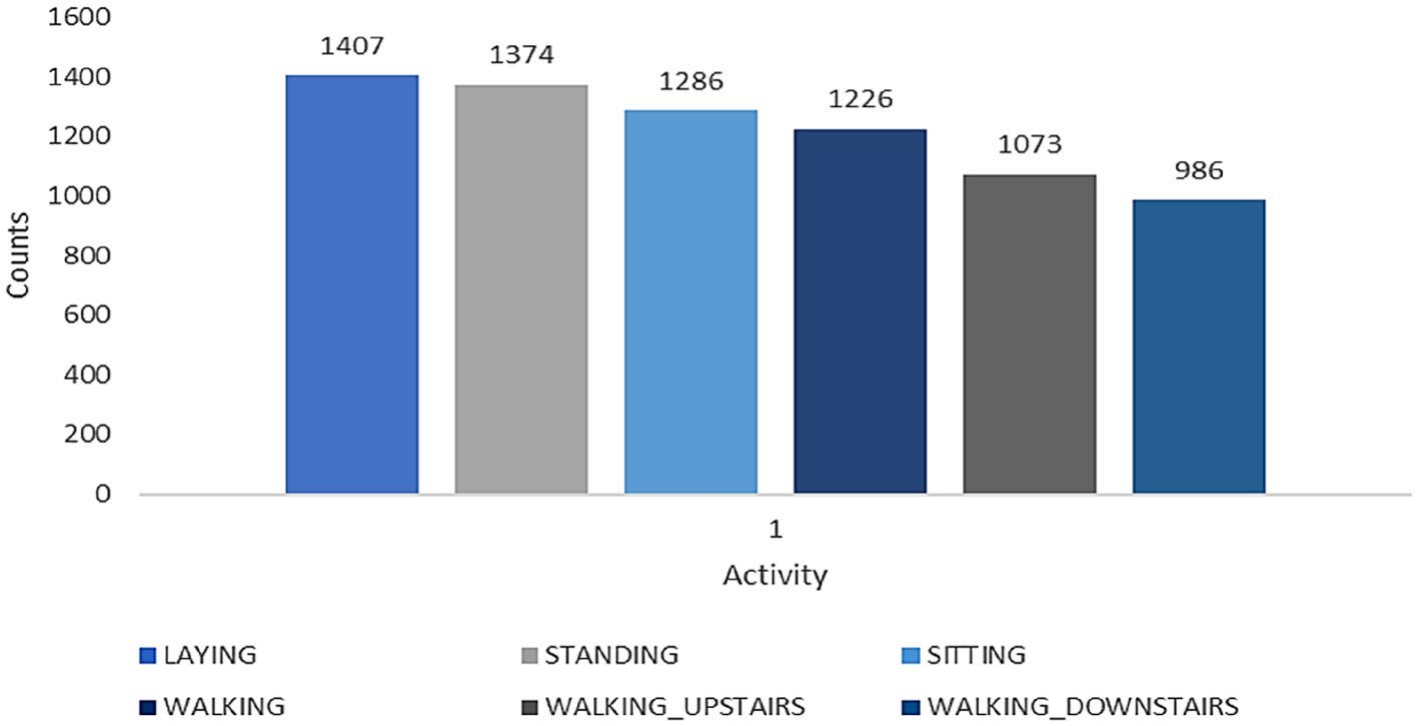
Human-activity-recognition-with-smartphones dataset.

The dataset human-activity-recognition-with-smartphones contains the activities of laying Standing, Sitting, Walking, walking upstairs, Walking downstairs. The number of data is predicted from the Fig. 4. The train and test data count in this dataset is 7352.

### Data cleaning and reduction

Dataset has been cleaned by removing duplicates, invalid values, and ensuring there is an even distribution of data for each activity. Data reduction was accomplished using well-known methods including principal component analysis and t-SNE. In order to select the most useful characteristics from a dataset, principal component analysis (PCA) can be performed to reduce the dimensionality of the original features. The principal component analysis (PCA) is an unsupervised technique (data without labelling) that uses the correlation between attributes to identify the patterns in the data. PCA is used to create a lower-dimensional subspace of features while still retaining the important features of the original feature set. Linear combinations of the features already present in the data set are used to create primary components, which are then used to describe the original data set. On the other hand, if a non-linear high-dimensional feature dataset is required to model the data, then modelling it with parameters generated by doing PCA on the data set will yield a very bad model, leading to less accurate recognition results. When this restriction is applied, data reduction using the t-SNE approach is a viable solution.

### t-SNE algorithm


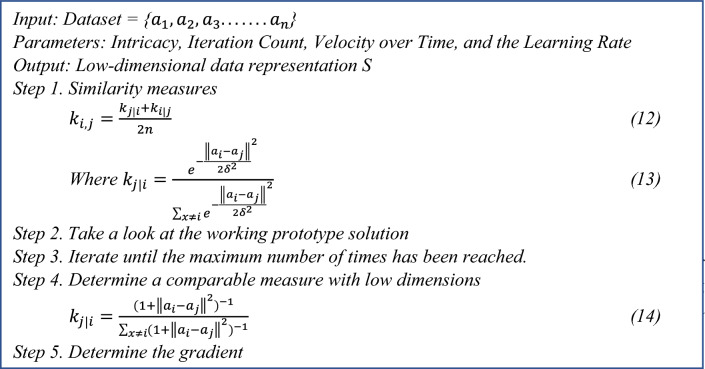


Additionally, a search strategy has been conducted across the following hyper-parameters for each framework, Searching (0.00001 to 0.004) with a 0.0005-point increase in the Learning Rate Number of samples: 16,32,64, Search range of training iteration, from 100 to 400 with a 100-step increment. The Adam optimizer is used in the proposed hybrid CNN model.

## Result and discussion

Several experiments have been carried out in order to evaluate the effectiveness of the improved CCN model that was provided earlier. Precision, Accuracy, Recall, and F-Measure are the parameters that are used to compare the result. These characteristics are determined:

15 $$Accuracy (Acc)=\frac{{T}_{pos}+{T}_{neg}}{{T}_{pos}+{T}_{neg}+{F}_{pos}+{F}_{neg}}$$

**Figure 5:**
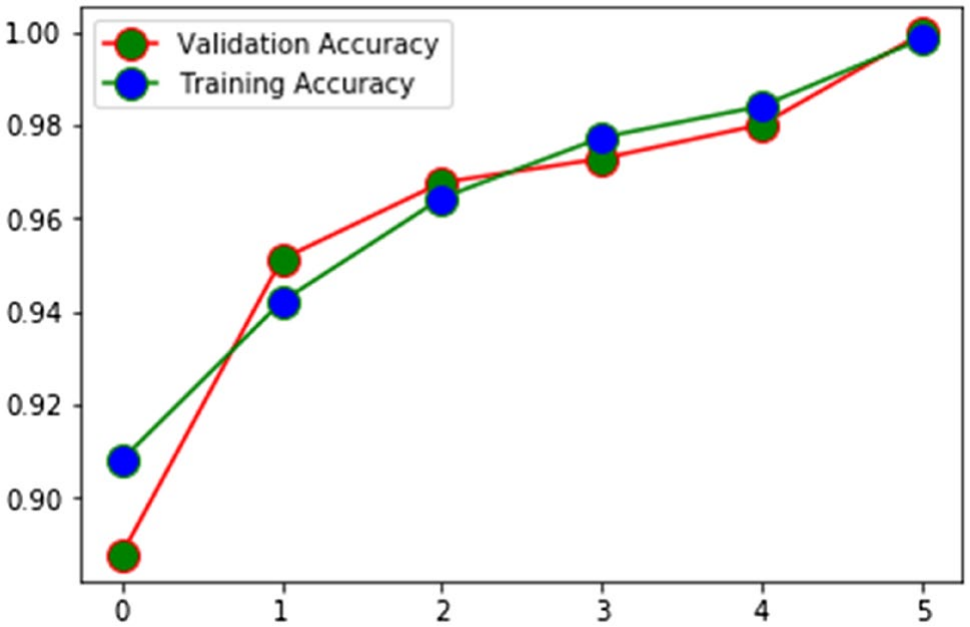
Training and validation accuracy of EHAM.

**Figure 6:**
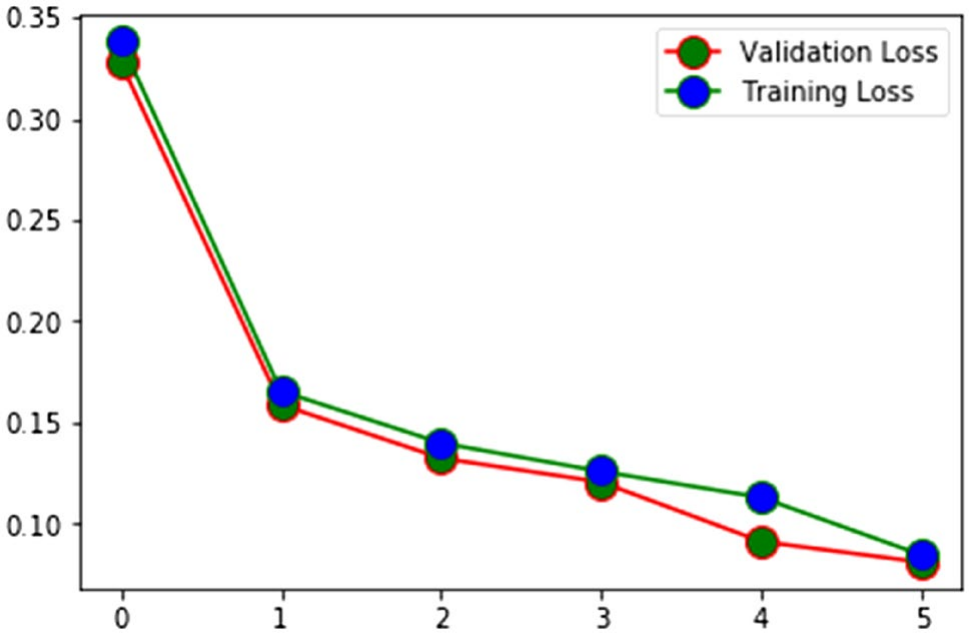
Training and validation loss of EHAM.

It’s a vote of confidence in the method being used to assess the HAR. The proportion of correctly categorized activities (identified) to the total number of classified activities is depicted in Figs. 5 and 6 and stands for accuracy. Sum of samples for which an identification was made is the recall, and percentage of correct identifications is the accuracy, both of which are stated in (16) and (17) for the HAM.

16 $$Precision (Pre)=\frac{{T}_{pos}}{{T}_{pos}+{F}_{pos}}$$

17 $$Recall (Re)=\frac{{T}_{pos}}{{T}_{pos}+{F}_{neg}}$$

Inadequate classifiers often have better classification accuracy, making this metric unreliable. Consequently, in addition to this criterion, another conventional factor known as F-measure is used. Measures of accuracy and recall are included in the F1 result, equating to the confidence level in the system’s ability to detect the agent’s actions. The F-measure is used to determine the reliability of samples of receive updates.

18 $$F1-Measure (F1M)=2\times \frac{Pre\times Re}{Pre+Re}$$

**Figure 7:**
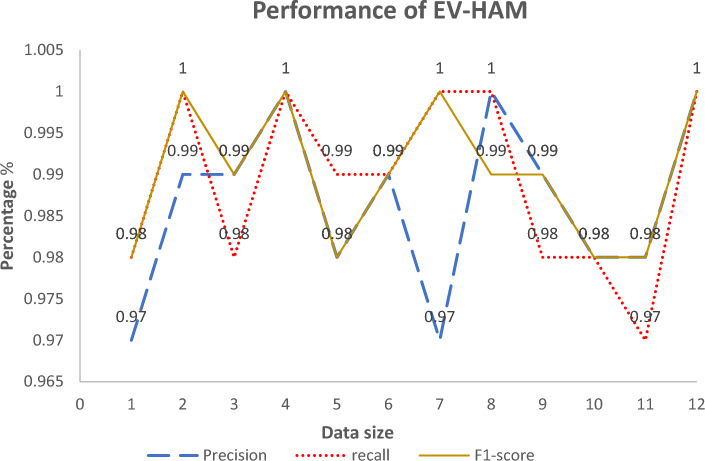
Graphical representation of performance evaluation of EV-HAM.

In this case, ${T}_{pos}$ stands for True Positives, ${T}_{neg}$ stands for ${T}_{neg}$, ${F}_{pos}$ stands for ${F}_{pos}$, and ${F}_{neg}$ is for False Negatives. For each dataset, accuracy was evaluated to determine the actual quality of the DHCNN (i.e., taking into account the entire collection of classes), and F-Measure, Precision, and Recall were computed to provide a more specific insight of how the DHCNN behaves when distinguishing a specific class. The Table 2 shows the results of performance measure of EV-HAM method. Graphical representation of performance evaluation shown in Fig. 7.

**Table 2:** Performance measure of EV-HAM.

	precision	recall	f1-score	support
1	0.97	0.98	0.98	35,800
2	0.99	1.00	1.00	46,327
3	0.99	0.98	0.99	111,285
4	1.00	1.00	1.00	59,486
5	0.98	0.99	0.98	60,744
6	0.99	0.99	0.99	63,492
7	0.97	1.00	1.00	31,606
8	1.00	1.00	0.99	21,709
9	0.99	0.98	0.99	124,392
10	0.98	0.98	0.98	21,353
11	0.98	0.97	0.98	17,385
12	1.00	1.00	1.00	6901
Accuracy			0.99	600,480
macro Avg	0.99	0.99	0.99	600,480
weighted Avg	0.99	0.99	0.99	600,480

**Figure 8:**
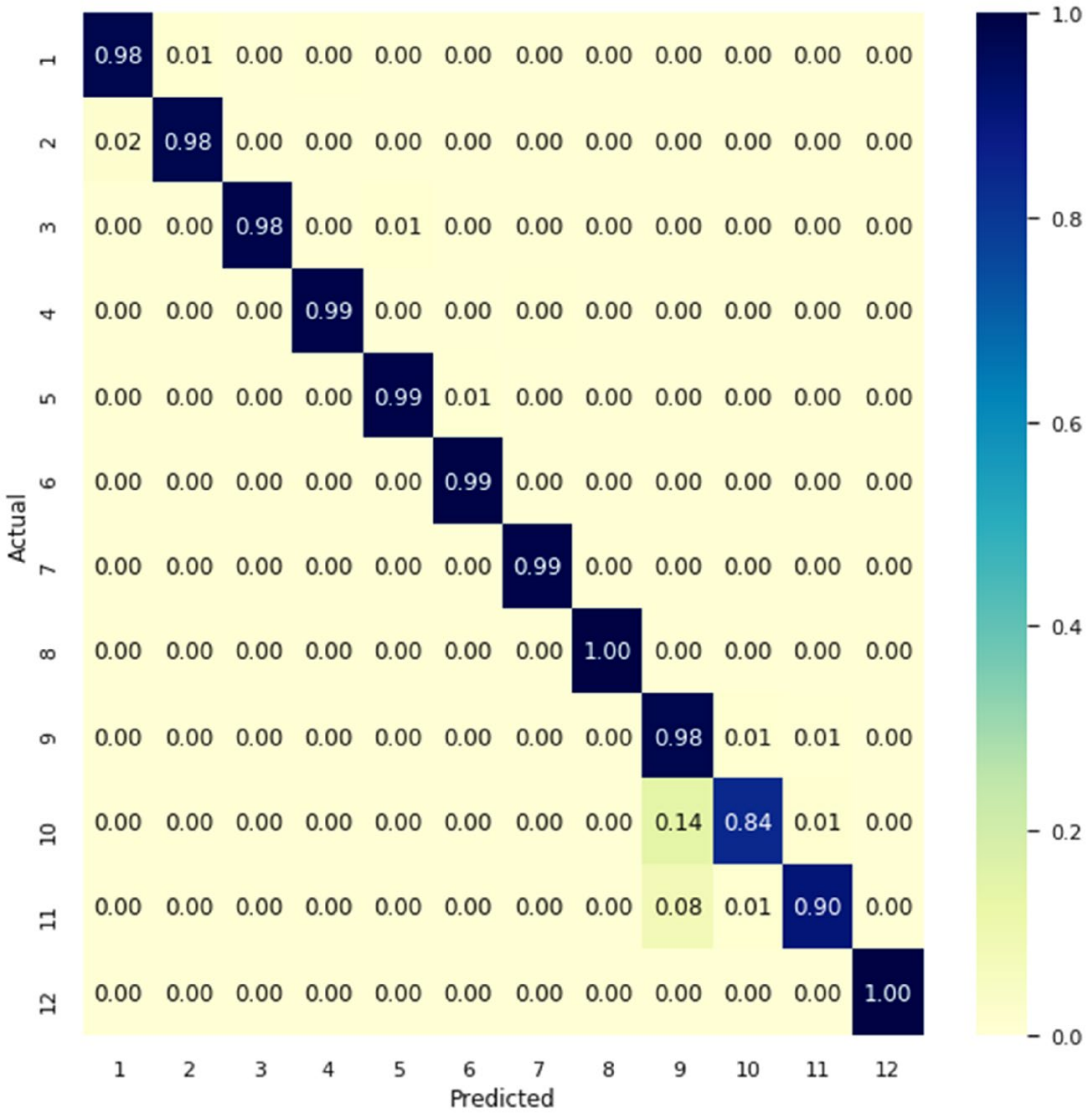
Confusion matrix of the publicly available dataset.

**Figure 9:**
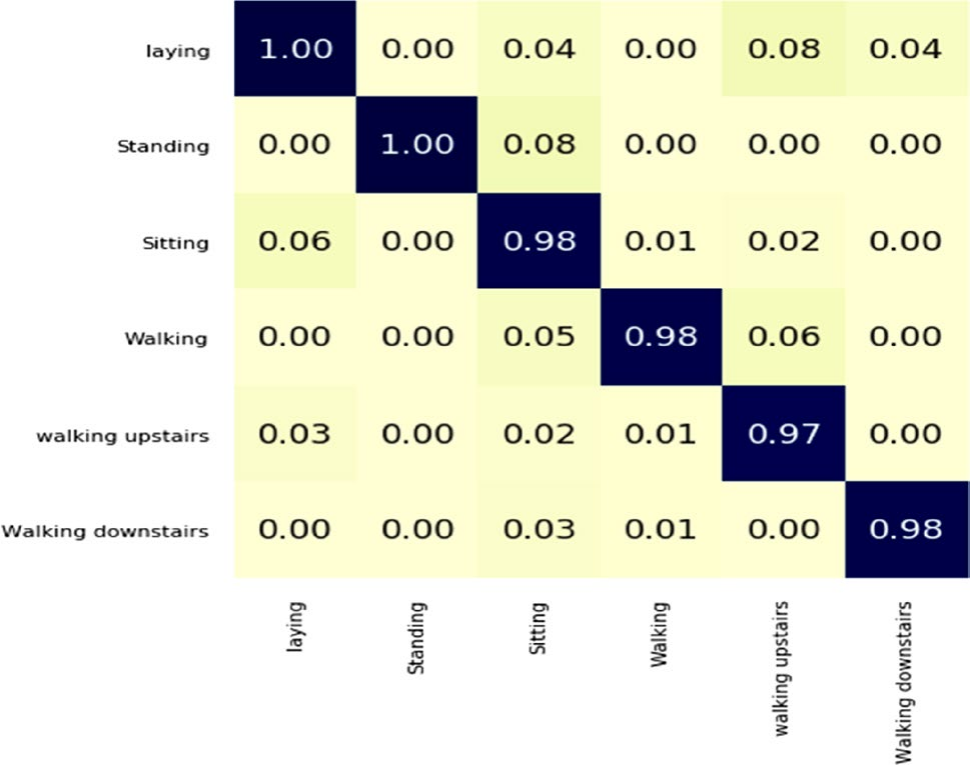
Confusion matrix of human-activity-recognition-with-smartphones dataset.

DHCNN algorithm confusion matrix is shown in Figs. 8 and 9. As can be seen in the blue diagonal cell of the confusion matrix the DHCNN classifier has an overall accuracy of 99%. The number of properly identified activities is represented in blue cells of the confusion matrix.

As an example, the top diagonal cell displays the number of correctly labelled walking scenarios. The memory rates for laying (100%) and standing (100%) are relatively high. The confusion matrix for the dataset human-activity-recognition-with-smartphones is shown in the Fig. 9.

**Figure 10:**
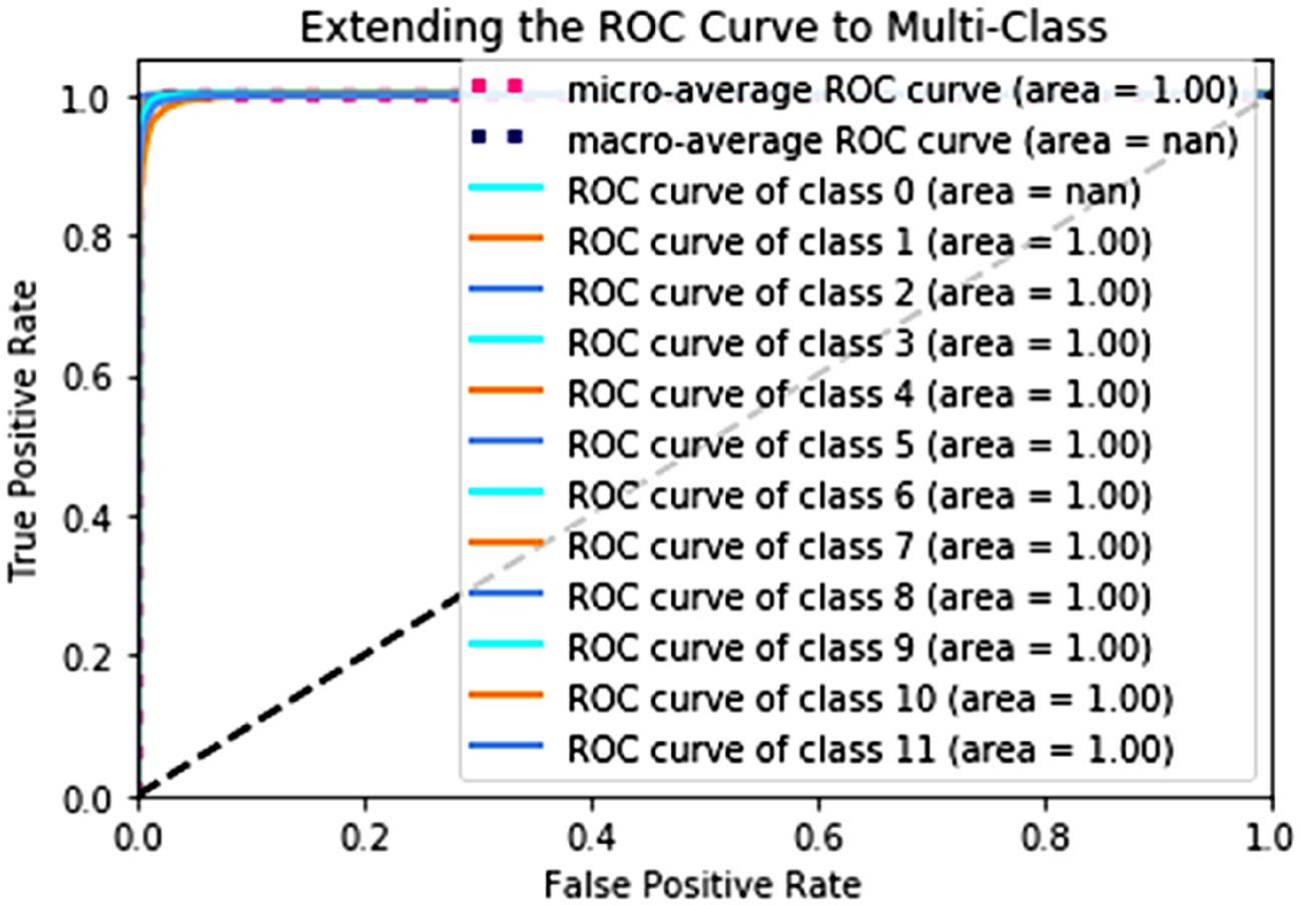
ROC of our Proposed model EV-HAM.

Figure 10 also displays the ROC curves for every class in the categorization, which helps to comprehend the model’s set of metrics. The ROC curve is a more reliable indicator of classification accuracy since it is unaffected by the uneven distribution of class labels in the sample.

The classifier’s true positive rate (recall) is compared to its false positive rate (sensitivity) in this graphic (fall-out). If a classifier were to randomly or poorly estimate its classes, its ROC curve would look like the diagonal dashed line in the graph. A further separation from the dashed line indicates that the classifier is more effective. All curves in a perfect classifier would meet in the upper left corner. Because of this less-than-ideal identification conclusion, the curves approached the edge but did not meet it, with the exception of the Laying curve, which had the highest individual classification accuracy (99%).

Our EV-HAM system takes advantage of a cloud situation via a wirelessly connected, wearable sensor, and a DHCNN model, and the resulting architecture meets all of the system’s needs. Here we conducted several tests to test number of tests to test out the efficacy of deep learning methods. We test these models on various proposed datasets containing image sequences and a variety of proposed datasets containing image sequences, and we report on their actual quality. With respect to accuracy across all frame sequences, our suggested DHCNN model outperformed all others. It uses a structured hierarchical semantic network to represent and organise technological concepts or domains. Then, it employs dual-link prediction techniques to identify and assess potential connections or relationships within this network^67,68^.

Table 3 shows the experimental outcomes of several deep-learning models. For example, the suggested HCNN obtained an average accuracy of 99%, while the MLP produced an accuracy of 85.25%, the CNN reached a precision of 87.65%, the LSTM acquired an accuracy of 82.85%, the Bi-LSTM had a precision of 89.5%, and so on as shown in Table 3.

**Table 3:** Performance comparison with baseline model.

Model	precision	recall	f1-score
MLP^62^	85.25	86	85.1
CNN^63^	92.4	92.0	91.6
LSTM^64^	82.85	83	82.3
Bi-LSTM ^61^	89.5	89	88.2
CNN-GRU^65^	90.2	90.5	90.0
Residual-BiLSTM^66^	93.2	93.1	93.0
DHCNN	99	1.0	99

While existing models only retrieve one sort of feature from the input data, the suggested model can learn both spatiotemporal information, which is able to learn both spatial and temporal information, which is what gives it its superior performance. Even though it takes more time with the basic structure, learning has accelerated. Requiring sensor fusion. These approaches require properly labelled data, yet they are effective for solving complex, large-scale HAM situations requiring sensor fusion. Processing time series information from sensors like accelerometers and gyroscopes is significantly more difficult than processing data from other sensors like cameras. It takes a lot of work for a specialist to properly divide up a task and identify it using a long time series. As a subset of parametric techniques, deep learning approaches boast both excellent accuracy and a lengthy training time complexity. Discoverable learning, which LSTM also employs, is the reason for its superior accuracy. The parameters to be acquired are very high because of the high computing complexity of the approach, but it is well-suited for complicated tasks and has great generalization power. Since RNNs have fewer parameters, they run faster, but LSTMs are more reliable.

## Conclusion and future work

In today’s healthcare system, HAR research is crucial. Researchers’ interest in this area has grown as a result of the growing requirement to evaluate time series data in order to HAR. In HAR, picking useful features from time series data is essential. There are many obstacles in this field, but there is also a requirement for a technique that accurately classifies actions. In this study, we present a novel Internet of Things (IoT) system for continuous, individual monitoring of routine domestic tasks. The technology combines a Wi-Fi wearable sensor with Deep Learning Methods to provide data on a wide range of actions, from which aberrant patterns can be inferred. The given method is intended to be scaled up to provide individualized data from a plethora of wearable sensors (as in a multi-occupant dwelling). This research proposes the entire information gathering to model generation workflow for human activity identification using Wi-Fi CSI. To learn how Wi-Fi settings, the placement of routers, and the surrounding environment affect CSI data, we conducted studies. We summarised our findings and suggested a network setup based on the data we were given. We presented a novel method of data gathering that is seamlessly woven into people’s regular routines, where several tasks can be completed in any order over the course of a limited length of time. Both the dataset and the data gathering methods are freely available for other researchers to utilise. Our final model is able to accurately categorise 12 activities from dataset1 and 6 activities from dataset2 with a 99% success rate. The preliminary experimental results show that the suggested ensemble algorithm outperforms the state-of-the-art algorithms in terms of classification accuracy. To further improve the approach’s performance, we plan to create a new hybrid feature selection technique using a genetic algorithm as part of our future study. This system’s architecture allows for the incorporation of complicated signal processing systems via the application of technologies to the development of compact, transportable, and self-sufficient integrated human recognition systems. In future elderly monitoring must be enhanced by video conferencing technology, so that originality of activity will be recognized clearly. For improving monitoring accuracy, IoT based architecture can be integrated.

## Data Availability

All data generated or analyzed during this study are included in this article. Access Details:The code can be accessed at [https://doi.org/10.5281/zenodo.10215757].

## Code availability

The Deep Hybrid Convolutional Neural Network (DHCNN) code, developed for the Elderly and Visually Impaired Human Activity Monitoring (EV-HAM) system in this study, is partially available to the public. The core components of this code, essential to the Wi-Sense system, can be accessed as required. Wi-Sense utilizes Wi-Fi channel state information (CSI) for accurate human activity identification, offering significant benefits to the elderly and visually impaired. Should the complete code be needed, requests can be directed to the corresponding author.

## References

[1] Serpush, F., Menhaj, M. B., Masoumi, B., & Karasfi, B. Wearable sensor-based human activity recognition in the smart healthcare system. *Comput. Intell. Neurosci*. (2022). 10.1155/2022/1391906 PMC8894054 35251142

[2] Nweke HF, Teh YW, Mujtaba G, Al-garadi MA (2019). Data fusion and multiple classifier systems for human activity detection and health monitoring: Review and open research directions. Inf. Fus..

[3] Janidarmian M, Fekr AR, Radecka K, Zilic Z (2017). A comprehensive analysis on wearable acceleration sensors in human activity recognition. Sensors.

[4] Zhang, R., *et al*. Differential Feature Awareness Network within Antagonistic Learning for Infrared-Visible Object Detection. In *IEEE Transactions on Circuits and Systems for Video Technology*, (2023).

[5] Yousefi S, Narui H, Dayal S (2017). A survey on behavior recognition using wifi channel state information. IEEE Commun. Mag. J..

[6] Gravina R, Alinia P, Ghasemzadeh H, Fortino G (2017). Multi-sensor fusion in body sensor networks: State-of-the-art and research challenges. Inf. Fus..

[7] Roy N, Misra A, Cook D (2016). Ambient and smartphone sensor assisted ADL recognition in multi-inhabitant smart environments. J. Ambient Intell. Hum. Comput..

[8] Singh D, Merdivan E, Hanke S, Kropf J, Geist M, Holzinger A, Learning TIM, Extraction K (2017). Convolutional and recurrent neural networks for activity recognition in smart environment. Towards Integrative Machine Learning and Knowledge Extraction.

[9] Lara OD, Labrador MA (2013). A survey on human activity recognition using wearable sensors. IEEE Commun. Surveys Tutor..

[10] Bulling A, Blanke U, Schiele B (2014). A tutorial on human activity recognition using body-worn inertial sensors. ACM Comput. Surveys (CSUR).

[11] Halperin D, Hu W, Sheth A, Wetherall D (2011). Tool release: Gathering 802.11N traces with channel state information. ACM SIGCOMM Comput. Commun. Rev..

[12] Chowdhury, T.Z. Using Wi-Fi channel state information (CSI) for human activity recognition and fall detection, Ph.D. dissertation, (University of British Columbia, Vancouver, 2018).

[13] Chang, R. Y., Liu, S., & Cheng, Y. Device-free indoor localization using Wi-Fi channel state information for Internet of things. In *IEEE Global Communications Conference (GLOBECOM)*, 1–7 (2018).

[14] Chen J, Li F, Chen H, Yang S, Wang Y (2019). Dynamic gesture recognition using wireless signals with less disturbance. Personal Ubiquitous Comput..

[15] Hassannejad, H. *et al*. Food image recognition using very deep convolutional networks. In *MADiMa 2016—Proceedings of the 2nd International Workshop on Multimedia Assisted Dietary Management, co-located with ACM Multimedia* 2016, 41–49 (2016).

[16] Miotto R, Wang F, Wang S, Jiang X, Dudley JT (2018). Deep learning for healthcare: Review, opportunities and challenges. Brief. Bioinform..

[17] Jiang, W., & Yin, Z. Human activity recognition using wearable sensors by deep convolutional neural networks. In *MM 2015—Proceedings of the 2015 ACM Multimedia Conference* 1307–1310 (2015).

[18] Zeng, M. *et al*. Convolutional neural networks for human activity recognition using mobile sensors. In *Proceedings of the 2014 6th International Conference on Mobile Computing, Applications and Services, MobiCASE 2014*, 197–205 (2015).

[19] Garcia PP, Santos TG, Machado MA, Mendes N (2023). Deep learning framework for controlling work sequence in collaborative human-robot assembly processes. Sensors.

[20] Chen, Y., & Xue, Y. A deep learning approach to human activity recognition based on single accelerometer. In *Proceedings—2015 IEEE International Conference on Systems, Man, and Cybernetics, SMC 2015*, 1488–1492 (2016).

[21] Yang W, Zhang J, Cai J, Xu Z (2023). HybridNet: Integrating GCN and CNN for skeleton-based action recognition. Appl. Intell..

[22] Hsi-Chiang, C., Yi-Ming, W., & Huai-Yuan, C. Design intelligent wheelchair with ECG measurement and wireless transmission function. In *Technology and Health Care 24.s1*, S345–S355 (2016). 10.3233/THC-151092 26444818

[23] Mritha, R., & Elanchezhian, C., *et al*. A better engineering design: low-cost assistance kit for manual wheelchair users with enhanced obstacle detection. J. Eng. Technol. Sci. **47**(4) (2015).

[24] Cho, J., Kim, J., & Kim, T. Smartphone-based human activity classification and energy expenditure generation in building environments. In *Proceedings of the 7th international symposium on sustainable healthy buildings*, 97–105 (2012).

[25] Matthew, B. *et al*. Poster: Gait-based smartphone user identification. In *Proceedings of the 9th international conference on Mobile systems, applications, and services*, 395–396 (2011).

[26] Zhou G, Zhang R, Huang S (2021). Generalized buffering algorithm. IEEE Access.

[27] Ahn, D., Kim, S., Hong, H., & Ko, B. C. STAR-Transformer: A spatio-temporal cross attention transformer for human action recognition. In *Proceedings of the IEEE/CVF Winter Conference on Applications of Computer Vision*, 3330–3339 (2023).

[28] Yang W, Zhang J, Cai J, Zhiyong Xu (2023). HybridNet: integrating GCN and CNN for skeleton-based action recognition. Appl. Intell..

[29] Noori FM, Uddin MZ, Torresen J (2021). Ultra-wideband radar-based activity recognition using deep learning. IEEE Access.

[30] Liang X, Huang Z, Yang S, Qiu L (2018). Device-free motion & trajectory detection via RFID. ACM Trans. Embed. Comput. Syst..

[31] Zhao Z, Xu G, Zhang N, Zhang Q (2022). Performance analysis of the hybrid satellite-terrestrial relay network with opportunistic scheduling over generalized fading channels. IEEE Trans. Vehic. Technol..

[32] Mi W, Xia Y, Bian Y (2019). Meta-analysis of the association between aldose reductase gene (CA)n microsatellite variants and risk of diabetic retinopathy. Exp. Ther. Med..

[33] Pan S (2022). A low-profile programmable beam scanning holographic array antenna without phase shifters. IEEE Internet of Things J..

[34] Li D, Ortegas KD, White M (2023). Exploring the computational effects of advanced deep neural networks on logical and activity learning for enhanced thinking skills. Systems.

[35] Hu Z (2023). Energy flow and functional behavior of individual muscles at different speeds during human walking. IEEE Trans. Neural Syst. Rehabil. Eng..

[36] Ding, G., Anselmi, N., Xu, W., Li, P., & Rocca, P. Interval-bounded optimal power pattern synthesis of array antenna excitations robust to mutual coupling. *IEEE Antennas Wire. Propagat. Lett*. (2023).

[37] Jiang, H. *et al*. Pa-count: Passenger counting in vehicles using wi-fi signals. *IEEE Trans. Mobile Comput*. (2023).

[38] Ma, K. *et al*. Reliability-constrained throughput optimization of industrial wireless sensor networks with energy harvesting relay. *IEEE Internet Things* J. **8**(17), 13343–13354 (2021).

[39] Li Q, Lin H, Tan X, Du S (2020). Consensus for multiagent-based supply chain systems under switching topology and uncertain demands. IEEE Trans. Syst. Man. Cybernet. Syst..

[40] Zhou D, Sheng M, Li J, Han Z (2023). Aerospace integrated networks innovation for empowering 6G: A survey and future challenges. IEEE Commun. Surveys Tutor..

[41] Lv Z, Wu J, Li Y, Song H (2022). Cross-layer optimization for industrial Internet of Things in real scene digital twins. IEEE Internet Things J.

[42] Qi, M. *et al*. Multi-region nonuniform brightness correction algorithm based on L-channel gamma transform. *Secur. Commun. Netw*. (2022).

[43] Cao K (2021). Achieving reliable and secure communications in wireless-powered NOMA systems. IEEE Trans. Vehic. Technol..

[44] Yan L, Shi Y, Wei M, Wu Y (2023). Multi-feature fusing local directional ternary pattern for facial expressions signal recognition based on video communication system. Alexandria Eng. J..

[45] Gao, Z. *et al*. Automatic interpretation and clinical evaluation for fundus fluorescein angiography images of diabetic retinopathy patients by deep learning. *British J. Ophthalmol*. 2022–321472 (2022). 10.1136/bjo-2022-321472 36171054

[46] Jin K (2023). MSHF: A multi-source heterogeneous fundus (MSHF) dataset for image quality assessment. Sci. Data..

[47] Ye X, Wang J, Qiu W, Chen Y, Shen L (2023). Excessive gliosis after vitrectomy for the highly myopic macular hole: A spectral domain optical coherence tomography study. Retina.

[48] Lu, S. *et al*. Soft tissue feature tracking based on deep matching network. *Comput. Model. Eng. Sci*. **136**(1), 363–379 (2023).

[49] Liu, C. *et al*. Robust online tensor completion for IoT streaming data recovery. *IEEE Trans. Neural Netw. Learn. Syst*. 2022. 10.1109/TNNLS.2022.3165076 35436201

[50] Guoqing Z, Weihao L, Xiang Z, Yizhi T, Gangchao L, Xianxing L, Ronghua D (2021). An innovative echo detection system with STM32 gated and PMT adjustable gain for airborne LiDAR. Int. J. Remote Sens..

[51] Zhou, G. *et al*. Gaussian inflection point selection for LiDAR hidden echo signal decomposition. *IEEE Geosci. Remote Sens. Lett*. 1–5 (2021).

[52] Gao, J. *et al*. MetaLoc: Learning to learn wireless localization. *IEEE J. Selected Areas Commun*. (2023).

[53] Shi J (2023). Adaptive waveform design utilizing an end-to-end learning-based pre-equalization neural network in a UVLC system. J. Lightwave Technol..

[54] Shi J (2023). Waveform-to-waveform end-to-end learning framework in a seamless fiber-terahertz integrated communication system. J. Lightwave Technol..

[55] Zhang Z, Guo D, Zhou S, Zhang J, Lin Y (2023). Flight trajectory prediction enabled by time-frequency wavelet transform. Nat. Commun..

[56] Lyu, T., Xu, H., Zhang, L., & Han, Z. Source selection and resource allocation in wireless powered relay networks: An adaptive dynamic programming based approach. *IEEE Internet Things J*. (2023).

[57] Cheng B (2017). Situation-Aware Dynamic Service Coordination in an IoT Environment. IEEE/ACM Trans. Netw..

[58] Tang, Y. *et al*. An improved method for soft tissue modeling. Biomedical signal processing and control, 65,2021.

[59] Liu Z, Feng J, Uden L (2023). Technology opportunity analysis using hierarchical semantic networks and dual-link prediction. Technovation.

[60] Guo, L., *et al*. Wiar: A public dataset for wifi-based activity recognition. *IEEE Access*. **7**, 154935–154945 (2019).

[61] Zhao Y, Yang R, Chevalier G, Xu X, Zhang Z (2018). Deep residual bidir-LSTM for human activity recognition using wearable sensors. Math. Probl. Eng..

[62] Nagpal D, Gupta S, Kumar D, Illés Z, Verma C, Dey B (2023). goldenAGER: A personalized feature fusion activity recognition model for elderly. IEEE Access.

[63] Hernández, F., Suárez, L.F., Villamizar, J.,& Altuve, M. Human activity recognition on smartphones using a bidirectional lstm network. In *Proceedings of the 2019 XXII Symposium on Image, Signal Processing and Artificial Vision (STSIVA), *1–5 (Bucaramanga, 2019).

[64] Shi, S., Wang, Y., Dong, H., Gui, G., & Ohtsuki, T. Smartphone-aided human activity recognition method using residual multi-layer perceptron. In *IEEE INFOCOM 2022-IEEE Conference on Computer Communications Workshops (INFOCOM WKSHPS)* 1–6 (2022).

[65] Wan S, Qi L, Xu X, Tong C, Gu Z (2020). Deep learning models for real-time human activity recognition with smartphones. Mob. Netw. Appl..

[66] Ullah, M., Ullah, H., Khan, S. D., & Cheikh, F. A. Stacked lstm network for human activity recognition using smartphone data. In *Proceedings of the 2019 8th European workshop on visual information processing (EUVIP)*, 175–180 (Roma, 2019).

[67] Syed Aziz, S. *et al*. Buried object sensing considering curved pipeline. In *IEEE Antennas and Wireless Propagation Letters*, 2771–2775 (2017).

[68] Bhavanasi G, Werthen-Brabants L, Dhaene T, Couckuyt I (2022). Patient activity recognition using radar sensors and machine learning. Neural Comput. Appl..

